# Would school closure for the 2009 H1N1 influenza epidemic have been worth the cost?: a computational simulation of Pennsylvania

**DOI:** 10.1186/1471-2458-11-353

**Published:** 2011-05-20

**Authors:** Shawn T Brown, Julie HY Tai, Rachel R Bailey, Philip C Cooley, William D Wheaton, Margaret A Potter, Ronald E Voorhees, Megan LeJeune, John J Grefenstette, Donald S Burke, Sarah M McGlone, Bruce Y Lee

**Affiliations:** 1 Graduate School of Public Health, University of Pittsburgh, 130 Desoto St., Pittsburgh, PA, 15261, USA; 2 Pittsburgh Supercomputing Center, Carnegie Mellon University, Pittsburgh, PA, USA; 3 School of Medicine, University of Pittsburgh, 200 Meyran Ave., Suite 200, Pittsburgh, PA 15213, USA; 4 Allegheny County Health Department, Pittsburgh, PA, USA; 5 Tepper School of Business, Carnegie Mellon University, Pittsburgh, PA, USA; 6 RTI International, Research Triangle Park, NC, USA

## Abstract

**Background:**

During the 2009 H1N1 influenza epidemic, policy makers debated over whether, when, and how long to close schools. While closing schools could have reduced influenza transmission thereby preventing cases, deaths, and health care costs, it may also have incurred substantial costs from increased childcare needs and lost productivity by teachers and other school employees.

**Methods:**

A combination of agent-based and Monte Carlo economic simulation modeling was used to determine the cost-benefit of closing schools (vs. not closing schools) for different durations (range: 1 to 8 weeks) and symptomatic case incidence triggers (range: 1 to 30) for the state of Pennsylvania during the 2009 H1N1 epidemic. Different scenarios varied the basic reproductive rate (R_0_) from 1.2, 1.6, to 2.0 and used case-hospitalization and case-fatality rates from the 2009 epidemic. Additional analyses determined the cost per influenza case averted of implementing school closure.

**Results:**

For all scenarios explored, closing schools resulted in substantially higher net costs than not closing schools. For R_0 _= 1.2, 1.6, and 2.0 epidemics, closing schools for 8 weeks would have resulted in median net costs of $21.0 billion (95% Range: $8.0 - $45.3 billion). The median cost per influenza case averted would have been $14,185 ($5,423 - $30,565) for R_0 _= 1.2, $25,253 ($9,501 - $53,461) for R_0 _= 1.6, and $23,483 ($8,870 - $50,926) for R_0 _= 2.0.

**Conclusions:**

Our study suggests that closing schools during the 2009 H1N1 epidemic could have resulted in substantial costs to society as the potential costs of lost productivity and childcare could have far outweighed the cost savings in preventing influenza cases.

## Background

During the 2009 H1N1 influenza epidemic, the Centers for Disease Control and Prevention (CDC) initially considered school closure as a mitigation intervention [1,2], and public health officials debated over whether, when, and how long to close schools [3,4]. Studies have suggested that a high degree of influenza transmission may occur in schools and sustained school closure may reduce the spread of both seasonal and epidemic influenza, thereby reducing morbidity and mortality [5-14]. However, school closure may be costly, as suggested by Sadique *et al. *[15] and Lempel, Hammond and Epstein [16] (an estimated $10 to $47 billion impact on the Gross Domestic Product (GDP) for 4 weeks of school closure in the U.S.), and may be a burden on parents (as elucidated in a recent CDC Morbidity and Mortality Weekly Report for the 2009 H1N1 epidemic [17]).

Therefore, there is a need to better understand the potential trade-offs between the costs and benefits of school closure during an epidemic similar to the 2009 H1N1 influenza from the perspectives of state and local decision-makers and society. To perform a cost-benefit analysis (CBA) of school closure during the 2009 epidemic, we developed an agent-based model (ABM) of the state of Pennsylvania to simulate the spread of influenza and the effects of school closure coupled with an economic model that translated the output from the ABM into costs.

## Methods

### State of Pennsylvania agent-based model (ABM)

The ABM represented each individual person living in the state of Pennsylvania and was similar in design to previously described models of Allegheny County, Pennsylvania [7,18], and the Washington, DC metropolitan area [19,20]. A geospatially explicit human agent database, termed a synthetic population, represented the state of Pennsylvania in the year 2000. Each agent was assigned to a household, so that at the census tract level the synthetic population contained realistic distributions of households, and agent demographics. This database comprised:

• synthesized households and persons,

• public and private K-12 schools, and

• workplaces including hospital and clinics.

**Figure 1:**
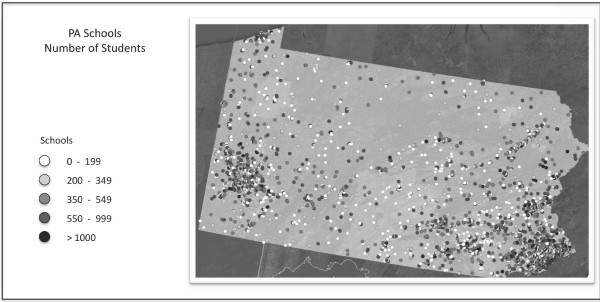
**The location and size of Pennsylvania schools**.

**Figure 2:**
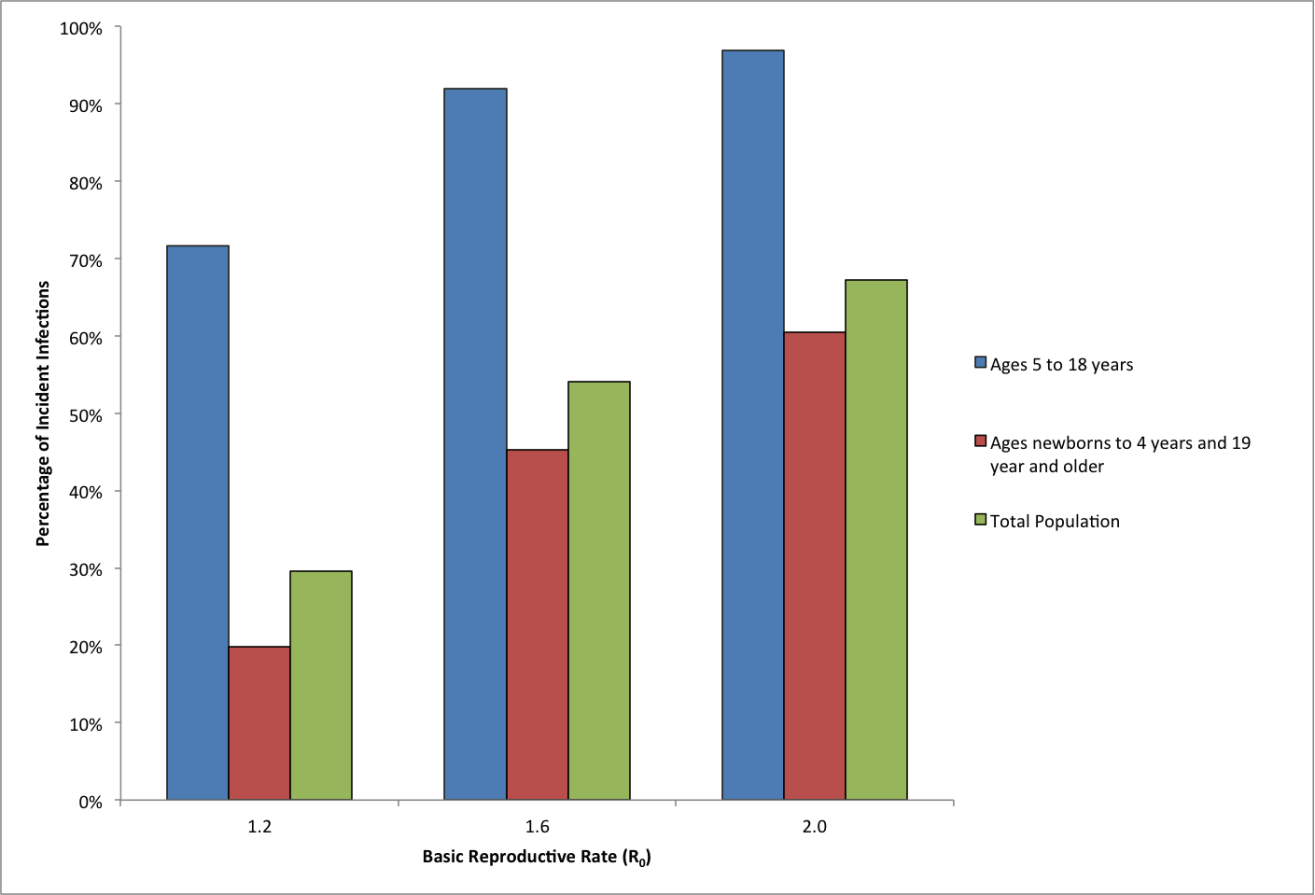
**Percent of selected populations infected for school-aged children, persons not going to school, and the total population in the state of Pennsylvania for the three R**_**0**_**'s explored**.

In addition school-aged agents were linked to the schools they attend and workers were linked to their workplace of employment (see [19] for additional details). Specifically, the synthetic population data included assignments of students and teachers to schools, facilitating simulation of school closure policies. Figure 1 shows the location and size of all of the schools in the state of Pennsylvania. The model was calibrated using empirical data from the 2009 H1N1 epidemic and historical epidemiologic studies of epidemic influenza. Important to this work and consistent with other influenza modeling efforts, schools are a critical place for the spread of the disease [21,22]. Figure 2 shows the percent of incident infections for school-aged children versus the rest of the population as a result of the model without school closures. It shows that across several basic reproductive rates, R_0 _(the average number of secondary infections produced by an infected individual in a completely susceptible population), that the percent of infections for school-aged children is much higher than that of others in the population. The US Census Bureau's 2005-2009 American Community Survey results show that there were insignificant changes to the age distribution in Pennsylvania between 2000 and 2009 [23].

To account for the spectrum of potential influenza transmission characteristics and dynamics, our simulation runs explored the effects of varying the R_0_, which is widely used as a measure of transmissibility. Estimates of the R_0 _for the 2009 H1N1 epidemic ranged from 1.2 - 1.7 [24-30]. Values of R_0 _equal to 1.2, 1.6, and 2.0 were explored in this study.

The school closure strategy modeled here is an individual school closure; implying that each school in the system is self-monitoring and closed for the specified duration when a certain in-school clinical incidence was reached (i.e. number of symptomatic cases detected in the school). In consulting with public health officials in Pennsylvania during the 2009 H1N1 pandemic, this scheme may be consistent with how school closure would be implemented in the State during an epidemic, and sensitivity analysis of other closure triggering mechanisms showed no significant change in the results. Our model varied two school closure policy parameters: the duration of closure (from 1 to 8 weeks) and the number of detected symptomatic cases that trigger a school to close (from 1 to 30 cases). These two parameters were varied independently to simulate different policy scenarios. School closure duration of 8 weeks have been shown in previous research to significantly decrease the overall attack rate [7], and longer school closure durations were not considered as they become logistically impractical to implement. Consistent with surveillance data collected during the 2009 H1N1 epidemic by the CDC, it is assumed that when the epidemic starts, schools are open. On weekends, students do not go to school and instead have increased activity in their neighborhoods and communities. The peak of the epidemic occurred well before possible extended holidays such as Thanksgiving and Christmas and so these were not considered.

For each scenario, the results presented are the average of 20 stochastic simulation runs, which is sufficient to obtain statistically significant results from computed confidence intervals. The simulations were all performed in parallel on the Intel Xeon based supercomputer, Axon, at the Pittsburgh Supercomputing Center and required approximately 15 minutes to complete using 20 compute cores. Hence, model outputs could be obtained very quickly in response to a crisis.

### Economic model

A Monte Carlo cost-benefit simulation model used the results from the ABM to estimate the cost-benefit of school closure in mitigating an influenza epidemic [31]. Table 1 lists the model's input parameters along with their distributions and sources. All costs and benefits were expressed in 2010 U.S. dollars. The model determined the net cost of implementing school closure using the following formula:(1)

**Table 1:** Key economic model inputs and distributions

Description (units)	Median	Source
Daily Wages*		[40]
Working parents/caregivers	$161.69 ($41.88-$345.70)	
Teacher	$212.12 ($103.23- $352.62)	
Other educational professionals	$336.12 ($190.25- $515.81)	
Durations		
Work hours per day	8	Assumption
Absenteeism from influenza (days)*	3.2 (1.85- 4.75)	[41]
Days of work missed per week of school closure	5	
Percent of infected individuals symptomatic	50%	
Probabilities/Ratios*		[42]
Student to teacher ratio	15 to 1	
Student to other education professional ratio	78 to 1	
Demographic Inputs		[43,44]
Percentage of caretaker households affected by school closure	71.5%	
Median number of persons per household under 18	1.9	
Case Fatality Percentage (95% CI)		[33]
Age 0-4	0.004% (0.001%-0.011%)	
Age 5-17	0.002% (0.000%-0.004%)	
Age 18-65	0.010% (0.007%-0.016%)	
Age 66-78+	0.010% (0.003%-0.025%)	
Outpatient Visit Probability (95% CI)		[45]
Age 0-4	0.455 ± 0.098	
Age 5-17	0.318 ± 0.061	
Age 18-65	0.313 ± 0.014	
Age 66-78+	0.620 ± 0.027	
Case Hospitalization Probability (95% CI)		[22]
Age 0-4	0.0033 (0.0021-0.0063)	
Age 5-17	0.0011 (0.0008-0.0018)	
Age 18-49	0.0015 (0.0011-0.0025)	
Age 65+	0.0016 (0.0010-0.0030)	
Outpatient Visit Costs (95% CI)		
Pediatric	$74.90	[46]
Adult	$104.77 ($69.14-$104.77)	[47]
Elderly	$155.92 ($118.39-$193.44)	[47]
Hospitalization Given Influenza (95% CI)		[48]
Age 1-17	$5,028 ($4,592-$5,464)	
Age 18-49	$6,506 ($6,071-$6,941)	
Age 50-64	$7,580 ($6,865-$8,295)	
Age 66+	$8,004 ($7,460-$8,548)	
Death Given Influenza (95% CI)	$7,129 ($5,347 - $9,296)	[49]

*All variables are gamma distributions approximated from normal distributions.

where a positive net cost meant that implementing school closure resulted in a net cost to society and a negative cost meant that implementing school closure resulted in net cost savings to society.

#### Cost of disease

The cost of disease was calculated as follows:(2)

Age-specific life expectancies were drawn from the Human Mortality Database [32] and a 3% discount rate adjusted future costs to 2010 values. To simulate conditions similar to the 2009 H1N1 epidemic, case hospitalizations and mortality rates from Presanis *et al. *[33] were used.

#### Cost of implementing school closure

The formula for computing the cost of school closure was as follows:(3)

The number of teachers and educational professionals per school were determined from student-teacher and student-educational professional ratios. It is assumed that all teachers and educational professionals were absent when the school was closed. To determine the number of parents affected by school closure, we used the following criteria. Children between the ages of 5 and 12 were defined to be school-aged children that could not care for themselves during school closure. Dual income and single parent families with only school-aged children might have needed to miss work or arrange for care during a school closure. We accounted for families with more than one school-aged child by dividing the results by the median number of persons under the age of 18 per family in Pennsylvania.

#### Cost per influenza case averted

In addition to the net cost, the cost per case averted of various school closure policies was computing using the formula:(4)

## Results

### Cost-benefit analysis (CBA)

**Figure 3:**
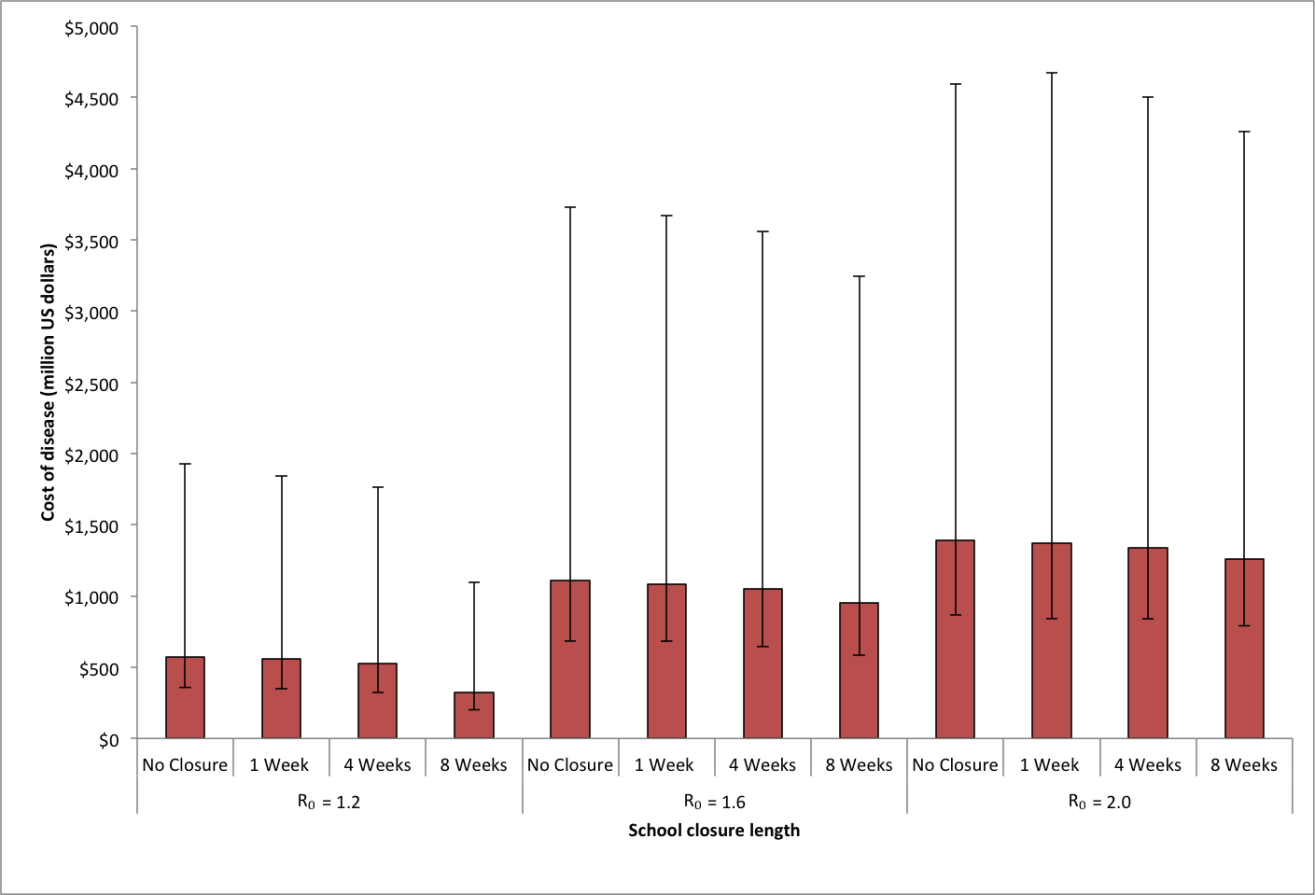
**Estimated cost of disease due to influenza for school closure policies of varying length for the three R**_**0**_**'s explored in the state of Pennsylvania (See Equation (2) in text)**. Error bars give the 5% and 95% distributions.

**Figure 4:**
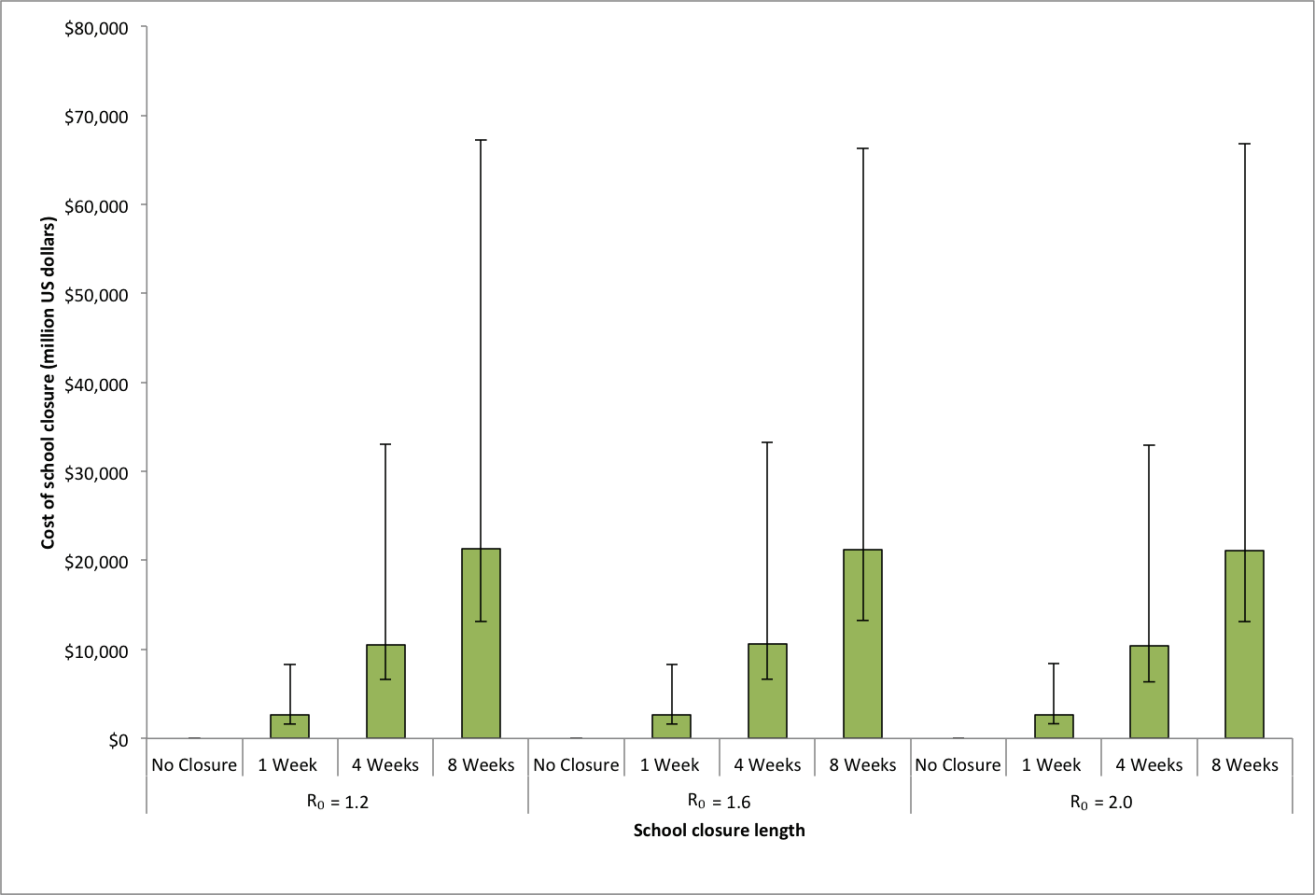
**Estimated costs due to school closure for policies of varying length for the three R**_**0**_**'s explored in the state of Pennsylvania (See Equation (3) in text)**. Error bars give the 5% and 95% distributions.

**Figure 5:**
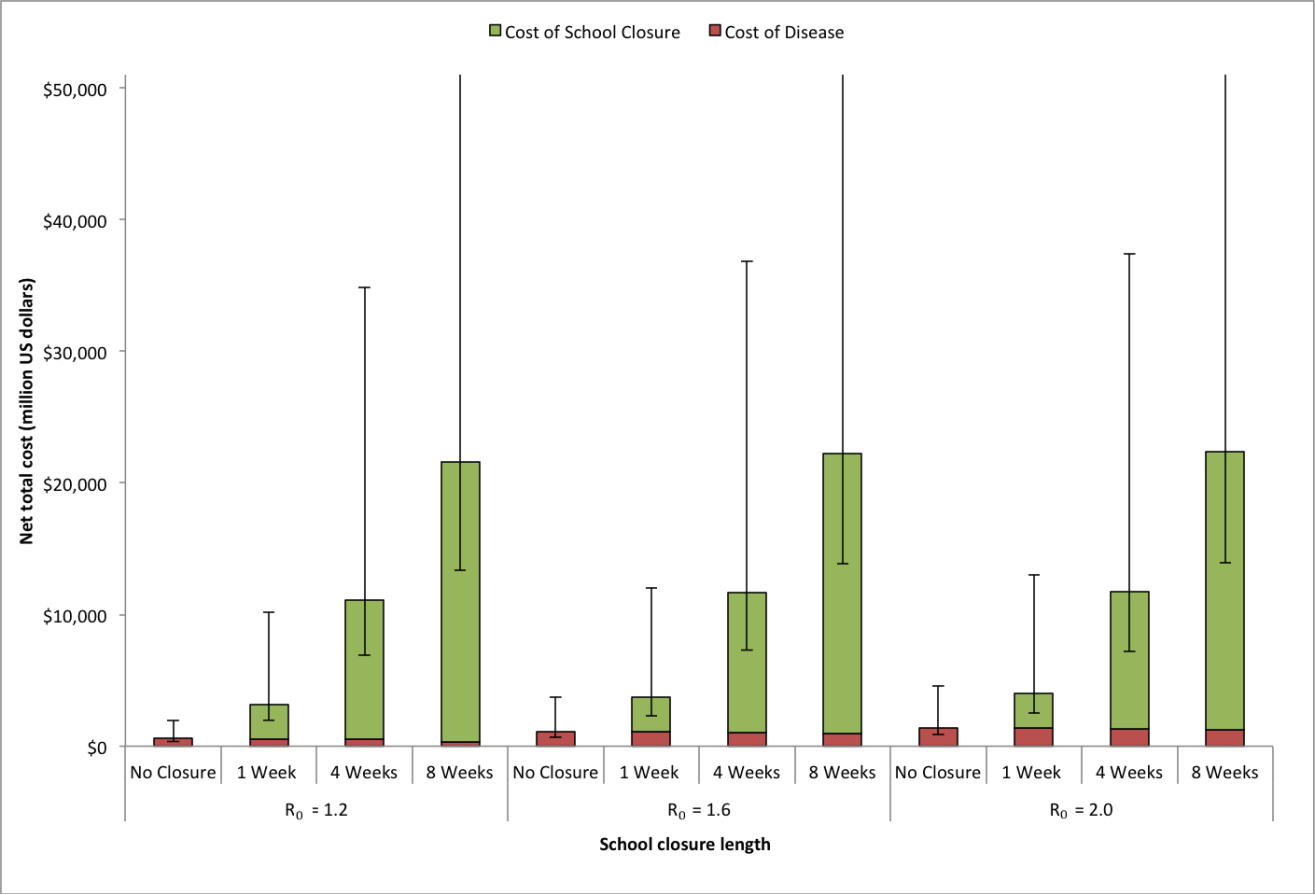
**Estimated total overall costs for school closure policies of varying length for the three R**_**0**_**'s explored in the state of Pennsylvania**. Error bars give the 5% and 95% distributions.

Figures 3, 4, and 5 present the cost of disease, cost of school closure and total costs as a function of school closure duration and the R_0 _of the epidemic for the state of Pennsylvania. As the R_0 _increased, the total number of influenza cases increased, which resulted in higher costs of disease (Figure 3). Consistent with a previous study, school closure of 1 to 4 weeks had a very modest effect on the epidemic. However, an school closure of 8 weeks in duration significantly decreased the number of cases [7]. With an 8 week school closure policy, the median cost of disease was $323.6 million (95% range: $122.0 - $734.7 million) for R_0 _= 1.2, $953.9 million (95% range: $370.5 - $2,290 million) for R_0 _= 1.6, and $1,263 million (95% range: $384.4 - $2,451 million) for R_0 _= 2.0. Varying the trigger by which schools decided to close (the number of symptomatic influenza detected in the school) had little effect on the cost of disease.

Figure 4 shows the cost of school closure with varying durations. Since the number of symptomatic cases detected in the school determined whether the school closed, varying the R_0 _could potentially have changed the number of schools that closed during an epidemic. However, this effect was not substantial, as the cost of school closure remained fairly constant across the entire range of R_0 _values explored. The median cost of school closure of 8 weeks was $21,277 million (95% range: $8,131 - $45,896 million) for R_0 _= 1.2, $21,248 million (95% range: $7,989 - $44,989 million) for R_0 _= 1.6, and $22,103 million (95% range: $7,973 - $45,697 million) for R_0 _= 2.0. The cost of school closure remained fairly constant across different numbers of symptomatic cases used to trigger school closure.

Figure 5 shows the total costs (the sum of the costs of disease and school closure) for various values of school closure durations and R_0_'s. It was notable that the cost of school closure contributed the vast majority of the total costs so that the cost of school closure primarily drove overall costs. The median total cost for an 8 week school closure policy was $21,600 million (95% values: $8,254 - $46,666 million) for R_0 _= 1.2, $22,201 million (95% values: $8,360 - $47,279 million) for R_0 _= 1.6, and $22,366 million (95% values: $8,445 - $48,696 million) for R_0 _= 2.0.

**Figure 6:**
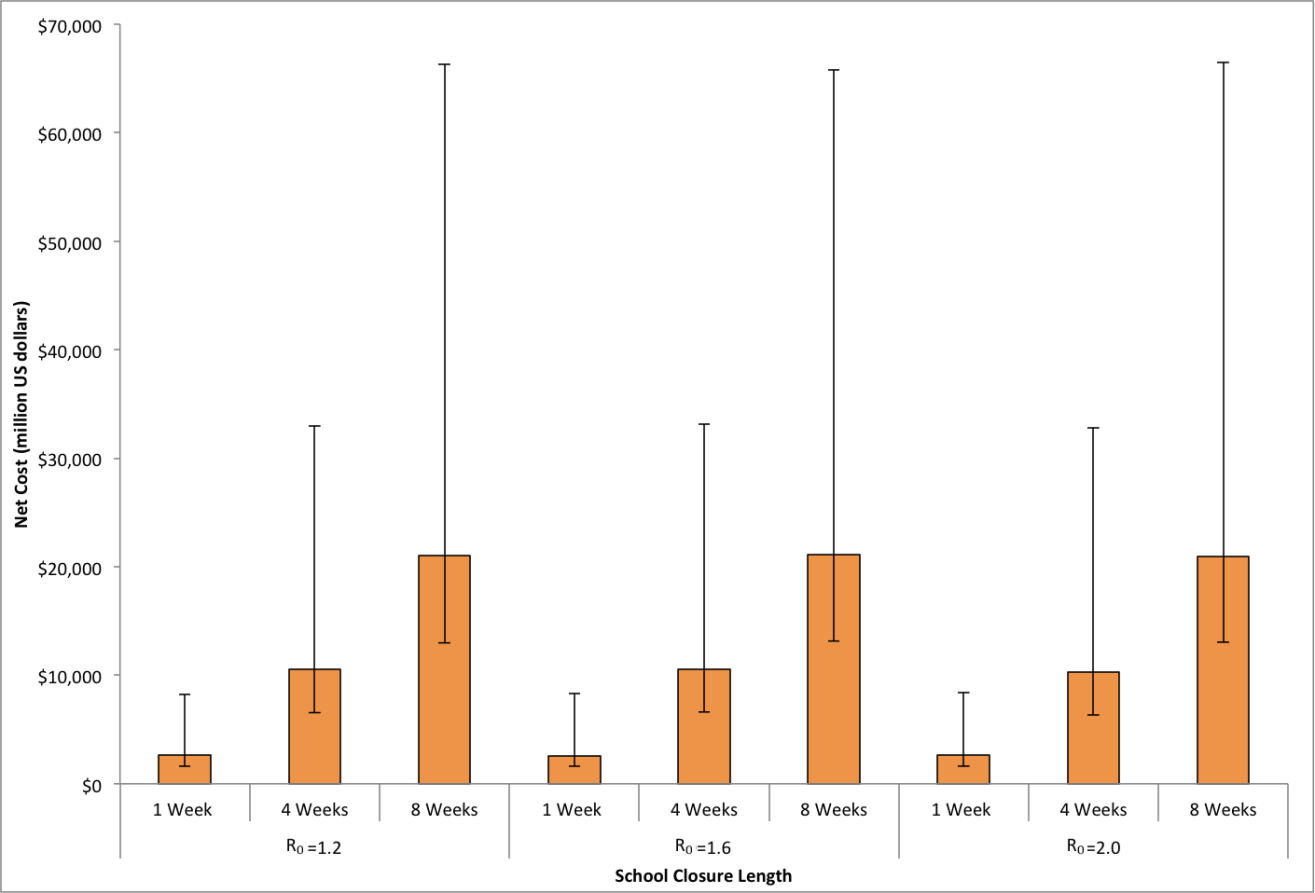
**Median net cost for school closure policies of varying length for the three R**_**0**_**'s explored in the state of Pennsylvania**. Error bars give the 5% and 95% distributions.

The net cost for the various durations of school closure and R_0_'s are shown in Figure 6. All of the scenarios explored exhibited net cost due to the substantially greater cost of the school closure versus the reduction in costs that resulted from mitigating the influenza epidemic. For school closures of 8 weeks duration, the median net cost was $21,028 million (95% values: $8,040 - $45,309 million) for R_0 _= 1.2, $21,093 million (95% values: $7,936 - $44,656 million) for R_0 _= 1.6, and $20,976 (95% values: $7,923 - $45,490 million) for R_0 _= 2.0.

**Figure 7:**
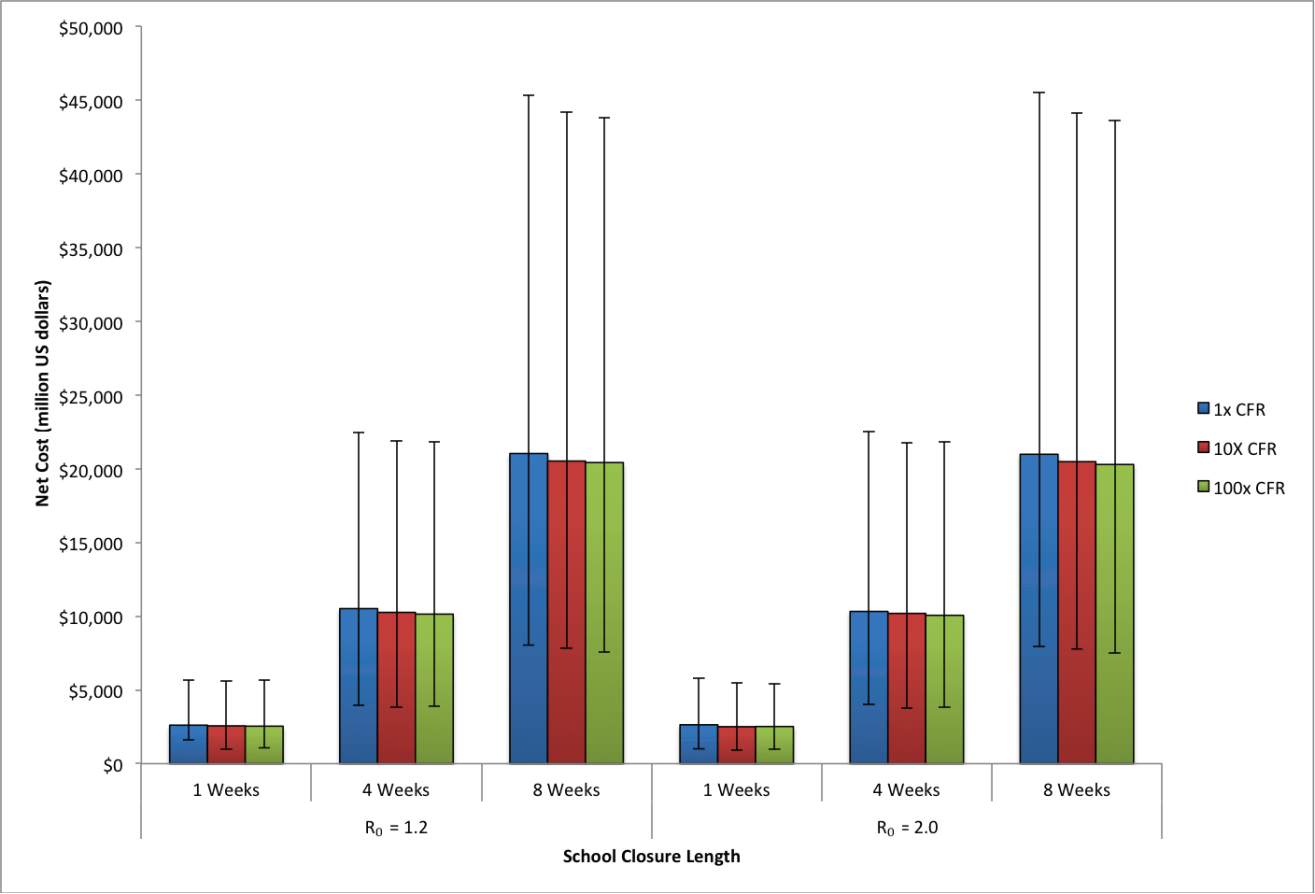
**Median net cost for school closure policies of varying length and case fatality rate (CFR)**. Error bars give the 5% and 95% distributions.

A potential factor to consider from the 2009 H1N1 epidemic is that the case fatality rate (CFR) was relatively low, especially compared with the estimates used in the preparedness planning for avian influenza [1]. To examine the potential sensitivity of the model to increased CFR, rates of 10 and 100 times that of the estimated CFR for 2009 H1N1 were explored. Figure 7 shows the net costs for varying durations of school closure with the 2009 H1N1 CFR as well as 10 and 100 times the original value. As expected, increases in CFR lower the net cost as the benefit of the school closure prevents mortality. This effect, however, is minor when compared to the cost of closing schools, and so the net costs do not drop significantly.

### Cost per influenza case averted

Table 2 shows the cost per influenza case averted for each of the scenarios explored. In the cases of R_0 _= 1.2 and 1.6, the cost per case averted increased from 1 week to 2 weeks of school closures as a consequence of the mitigation not having been effective. For school closure of 2 weeks and longer, and 1 week or longer in the case of R_0 _= 2.0, there was a decrease in the cost per case averted since longer school closures resulted in increased mitigation of the epidemic. The median cost per case averted for school closure of 8 weeks was $14,185 (95% values: $5,423 - $30,565) for R_0 _= 1.2, $25,253 (95% values: $9501 - $53,461) for R_0 _= 1.6, and $23,483 (95% values: $8,870 - $50,926) for R_0 _= 2.0.

**Table 2:** Cost per case averted for various school closure policies and R_0_'s

Closure Length (5 Case Symptomatic Incidence Trigger)					
R_0_	1 Week	2 Weeks	4 Weeks	8 Weeks	

1.2	$33,926 ($12,974 - $73,358)	$46,934 ($17,557 - $101,758)	$45,306 ($17,045 - $96,623)	$14,185 ($5,423 - $30,565)	
1.6	$68,077 ($25,734 - $148,201)	$73,734 ($28,233 - $159,194)	$45,823 ($17,027 - $98,276)	$25,253 ($9,501 - $53,461)	
2.0	$95,602 ($36,575 - $210,779)	$65,994 ($24,951 - $143,562)	$24,963 ($9,649 - $54,463)	$23,483 ($8,870 - $50,926)	

Symptomatic Incidence Trigger (8 Weeks School Closure Length)					

R_0_	1 Cases	3 Cases	10 Cases	20 Cases	30 Cases

1.2	$11,260 ($4,250 - $24,477)	$14,546 ($5,668 - $31,603)	$12,055 ($4,578 - $25,874)	$11,127 ($4,271 - $23,999)	$11,690 ($4,466 - $25,254)
1.6	$47,303 ($17,586-$100,794)	$30,858 ($11,658 - $66,629)	$19,477 ($7,474 - $41,922)	$17,269 ($6,429 - $36,726)	$18,113 ($6,990- $38,322)
2.0	$23,867 ($8,851 - $52,132)	$22,714 ($8,678 - $49,149)	$26,069 ($9,756 - $55,316)	$29,955 ($11,319 - $65,801)	$34,530 ($12,964 - $73,368)

The cost per case averted varied in surprising ways depending on the trigger for closure. For example, it was shown in previous work simulating school closure for Allegheny County, that increasing the number of detected symptomatic cases needed to trigger a school closure could have a positive effect in reducing the number of total infections during an epidemic [7]. There appeared to be a dependence on timing the school closure mitigation so that it spanned the peak of the epidemic in order to gain the maximum mitigation benefit, which resulted in a decrease in the number of cases as the trigger parameter was increased. Once the optimal number of triggering cases was reached, any further increases in the trigger yielded decreasing benefit. In the cost per case averted, we saw a similar trend when R_0_'s of 1.6 and 2.0 were simulated, which had optimal median costs at 20 trigger cases ($17,269) and 3 trigger cases ($22,714) respectively. For an epidemic with an R_0 _of 1.2, allowing schools to close when there was one symptomatic case produced a low cost per case averted, with a median cost of $11,260 per case. In this instance, the epidemic curve was relatively broad which indicated a slower build up to the peak of the epidemic. Closing schools when there was one symptomatic case detected allowed mitigation to have a substantial effect prior to the influenza taking significant hold in the population. At a trigger of 3 cases there was an increase in the cost per case averted; the cost per case averted trended downward until 20 cases.

## Discussion

During the 2009 H1N1 epidemic, using school closure as a sole mitigation strategy for epidemics may have been a burden to parents needing to provide childcare and miss work, as well as to teachers and other educational professionals. From our analysis, each day of school closure may have cost an estimated average of $120,000 per school in the state of Pennsylvania. The costs of school closure may have been approximately 5 to 40 times higher than the total costs from influenza without school closure mitigation, and therefore may have resulted in a net cost. Pennsylvania is a fairly representative state on which to perform modeling of school closures, having two major cities, Philadelphia and Pittsburgh, as well as a large rural area, and several smaller cities and towns. The results reported here are expected to be similar for other states and for the US as a whole.

These results indicate that school closure could have incurred a significant cost per case averted. By comparison, vaccination as an influenza epidemic mitigation strategy has been estimated to have a cost per case averted at less than $100 [34-36]; orders of magnitude less than the cost per case averted for even the most effective school closure policy. These results support the conclusion that the cost of closing schools versus the benefit of mitigating the 2009 H1N1 epidemic may have been too great to be a viable strategy. As the 2009 H1N1 epidemic had relatively low mortality rates, the net costs and cost per case averted could have changed significantly in the face of greater mortality. For CFR of up to 100 times that of 2009 H1N1, school closure still exhibits a net cost.

Merging large-scale epidemic models of school closure and economic models is the next logical step in understanding the relative advantages and disadvantages of school closure for epidemic mitigation. Prior studies have shown the epidemiologic benefits of school closure [7], and others have focused on the potential cost of school closure [15,16]. Our study merges these two approaches and provides a tool for decision makers to use to evaluate the potential cost-benefit trade-offs involved.

It is important to note that school closures may not have occurred in isolation. During the early part of the 2009 H1N1 epidemic, vaccines were not yet available and school closure and other non-pharmaceutical interventions may have been the only viable options. Combining different intervention strategies for mitigation (known as targeted-layered containment) and has been studied for epidemic influenza by Halloran *et al *[37]. School closure is a mitigation strategy that is targeted primarily to school-aged children and may be combined with interventions that target other age cohorts to yield a more effective intervention strategy. These results are meant to highlight an important factor in deciding whether to implement such a policy, but not to preclude it all together.

While our model shows net costs for any of the school closure scenarios explored, it does not include all of the factors that may be important to making the decision to close schools. Inherently the results are presented in terms of dollars and cents, and we do not purport any other judgments as to the value of mitigating epidemic influenza cases and deaths. Klaiman, Kraemer, and Stoto recently performed a search of media sources and determine that there are primarily four rationales for closing schools during an epidemic: limiting spread of the virus in the community, protecting vulnerable children, reacting to staff shortages or children kept at home because of parents' fear of infection [14]. In August of 2009, the CDC school closure guidance offered that local authorities should weigh "the risks of keeping the students in school with the disruption that school dismissal can cause" [2]. The economic component of this recommendation is explored here, and there may be many other considerations that affect the decision to close schools.

So, how could state and local officials use these results in deciding whether or not to close schools in the face of influenza epidemic? Officials need to balance the benefit of reducing the spread of influenza against what could be a substantial economic burden as a result of the closure. Moreover, the results of the ABM here and in previous work modeling Allegheny County [7] indicate that for closure to have an impact, the closure policy will need to be in effect for at least 6 to 8 weeks. Indeed the cost per case averted drops as school closures are extended to 8 weeks in length. Closing schools long enough to have a substantial effect on the disease spread is likely to be quite expensive, so officials should carefully weigh all of the options for mitigation before implementing a school closure policy.

### Limitations

All computer models are simplifications of reality and cannot account for every possible factor or interaction [38,39]. Rather than dictate courses of action, models do provide valuable information to decision makers about possible scenarios and relationships. An influenza epidemic and the resulting circumstances may not necessarily conform to the data and assumptions in any given model. Our model drew from referenced sources and previously published models. The economic model focused on health care costs and productivity losses directly resulting from absenteeism and mortality and did not include other possible negative externalities (e.g., lack of school lunch programs during school closure). Additionally, wages are an often used but imperfect proxy for productivity lost. The economic model does not include an attempt to account for persons who may be able to work-from-home, which may reduce the productivity loss estimates of school closure but is unlikely to change to conclusions of this research.

## Conclusions

If school closure had been widely used as a mitigation strategy for the 2009 H1N1 epidemic in Pennsylvania, the costs of school closure may have far outweighed the potential cost-savings from reducing the number influenza cases. As in previous studies, closing schools for at least 8 weeks is necessary to have an effective mitigation of an influenza epidemic. These findings may have applied over a wide range of R_0_'s and school closure policies for the state of Pennsylvania given the relatively low mortality rate of 2009 H1N1 and while isolating school closure from other possible interventions that might have been combined during the epidemic. Decision makers should carefully consider the possibility of substantial costs from increased child-care needs and lost productivity by parents, teachers and other school employees before implementing school closure.

## Competing interests

The authors declare that they have no competing interests.

## Authors' contributions

STB, JHYT, RRB, ML, and BYL were responsible for conceptualizing and designing the study, conducting the experiments, analyzing the results, and drafting of the manuscript. PCC and WDW contributed to the design of the agent-based model. MAP, REV, JJG, SMM, and DSB assisted in designing the experiments and interpreting the results. All authors reviewed, contributed to, and approved the final manuscript.

## Pre-publication history

The pre-publication history for this paper can be accessed here:

http://www.biomedcentral.com/1471-2458/11/353/prepub
